# Increased Needle Visibility in Ultrasound-Guided Percutaneous Liver Biopsy by an Echogenic Sheath: A Proof of Concept Study in a Human Cadaver

**DOI:** 10.1007/s00270-021-02783-8

**Published:** 2021-02-24

**Authors:** Jana S. Hopstaken, Leon de Jong, Jurgen J. Fütterer

**Affiliations:** 1 Department of Medical Imaging, Radboud University Medical Center, Geert Grooteplein 10, 6525 Nijmegen, GA The Netherlands; 2 Department of Surgery, Radboud University Medical Center, Nijmegen, The Netherlands; 3 Radboud Institute for Health Sciences, Radboud University Medical Center, Nijmegen, The Netherlands

**Keywords:** Liver biopsy, Ultrasound guided, Echogenicity, Needle tip visualization

## Abstract

**Purpose:**

For the safety and success of an ultrasound-guided percutaneous liver biopsy, needle visibility and needle tip identification are critical. The aim of this pilot study was to evaluate the influence of an innovative echogenic sheath placed over a standard biopsy needle on needle visibility in ultrasound imaging.

**Materials and methods:**

Ultrasound videos of three sheaths with different coating characteristics (echogenicity) and one conventional liver biopsy needle were recorded at two angles (30° and 60°) and two depths (5 and 10 cm) in a human cadaver. The videos were blinded for needle type and presented to five independent radiologists who used Likert-scale scoring to rank each video for six characteristics on needle visibility. In addition, a phantom model was used to acquire standardized images for quantitative evaluation of the ultrasound visibility. Comparative statistical analysis consisted of a one-way ANOVA.

**Results:**

The three prototype sheaths were ranked higher than the control needle at 60° with 5 cm depth, with an equal performance for the other conditions. The radiologists expressed more confidence in taking a biopsy with the echogenic sheaths than with the control needle, with 1 Likert score difference at 30°. Contrast analysis in the phantom model showed a statistically significant effect of a sheath (*p* = 0.004) on echogenic intensity.

**Conclusion:**

This pilot study suggests that the use of an echogenic sheath may increase needle visibility, particularly for trajectories requiring steeper insertion angles. To investigate the superiority of the echogenic sheath over conventional needles, a clinical study is necessary.

## Introduction

An image-guided percutaneous liver biopsy (PLB) is a regularly performed outpatient procedure. A biopsy is required to determine the nature of a lesion and is an essential prerequisite for constructing an appropriate treatment plan. PLB is considered a safe procedure [1] with a relatively low complication risk of 1.2–1.6% [2, 3]. It is generally performed under ultrasound guidance. The lack of ionizing radiation, real-time imaging and the wide availability are advantages of ultrasonography in PLB compared to computed-tomography (CT). A disadvantage, however, is that in tissue with high echogenicity or in case needles are inserted in substantial depth or at steep angles relative to the probe, needle identification can become challenging [4–6]. For the safety and success of a PLB, it is crucial that needle visibility and identification are optimal. These are also of importance in other procedures, such as those involving vascular access [7, 8] or in ultrasound-guided regional anesthesia [5]. To overcome poor needle visibility, two solutions are available: the imaging techniques may be adjusted or the visibility of the needle itself may be improved by increasing its echogenicity, i.e., the ability to reflect the ultrasound signal. The current commercially available needles try to optimize needle visibility by means of sandblasting, dimpling, or with a polymeric coating [4]. To provide insight in which of these needles is the most promising, the NICE study undertook descriptive bench-top testing and blind comparison of 10 echogenic needles [9]. A prototype needle with a polymeric coating, Sono-Coat™ (Encapson B.V, Enschede, The Netherlands), was ranked significantly higher than the others. However, these needles were tested in a phantom model and had fine-needle aspiration as intended use.

This preclinical pilot study aimed to evaluate ultrasound visibility of three different Sono-Sheaths™, a polymeric sheath with an echogenic tip placed over a conventional biopsy needle, in a human cadaver compared to a conventional biopsy needle. Primary endpoint was the qualitative performance on needle visibility expressed in ranking by radiologists, the secondary endpoint was the needle visibility expressed in quantitative metrics.

## Materials and Methods

### Ethical Consideration

For this study, no ethical approval was required. A male human cadaver was provided by the Department of Anatomy of the Radboud University Medical Center, Nijmegen, The Netherlands.

### Cadaver Model, Control and Study Needles

**Fig. 1:**
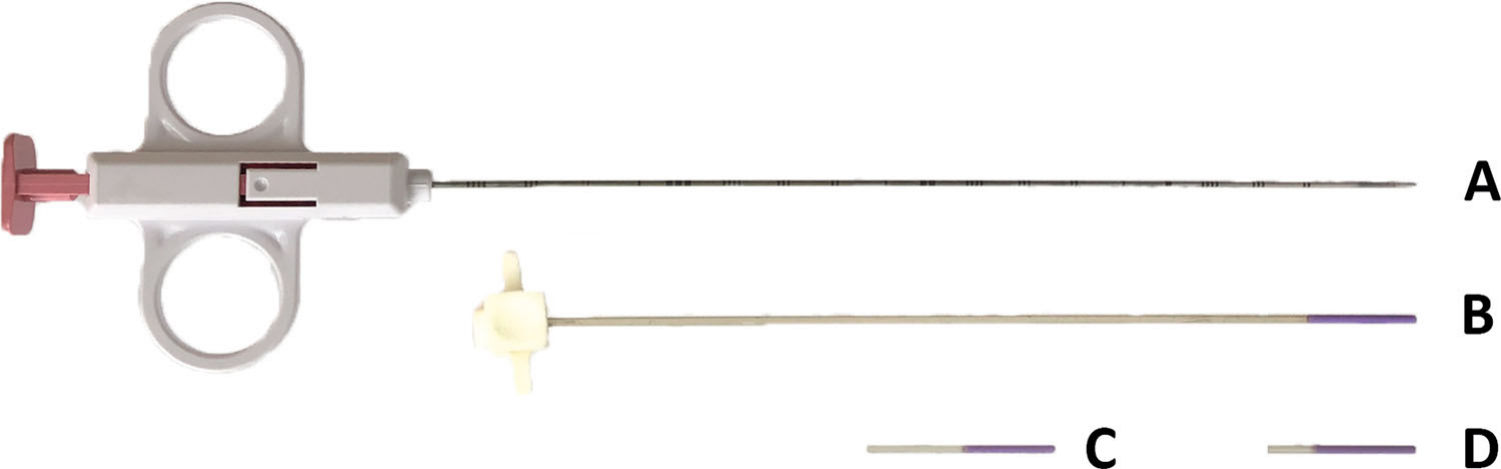
Control needle **A** and three Sono-Sheath™ prototypes (**B** = 1,**C **= 2,**D** = 3)

The cadaver was a defrosted fresh-frozen cadaver (i.e. no embalmment). The study material consisted of one control needle and three different prototypes. The control needle, the Argon SuperCore™ Semi-Automatic Biopsy Instrument, 18 G, 20 cm (Argon Medical Devices, Frisco, TX, USA), is the most commonly used biopsy needle in our institute. It has an echogenic tip on the outer cannula by means of sandblasting. The three prototypes consisted of three Sono-Sheaths™ (Encapson BV, Enschede, The Netherlands). These are polymeric sheaths with Sono-Coat™, an echogenic coating at the tip. The three Sono-Sheath™ prototypes were each coated with a different version of Sono-Coat™. This technology is based on a polymer coating matrix containing acoustically reflective microspheres, which scatter the ultrasound waves in all directions. This scattering effect causes more ultrasound signals to be reflected to the probe. By varying the size and surface density of the microspheres, the reflectivity of the coating can be optimized to give the clearest image for the depth of the procedure. The higher the reflectivity the less delineated the image. Sheath 1 is designed to be less reflective and to give the most delineated image, sheath 2 is a balance between reflection and delineation, and sheath 3 is designed to provide the highest reflection, with the risk of generating artifacts. The sheaths are designed to be placed over a biopsy needle. During the procedure, the sheaths were therefore placed over the control needle (Fig. 1) and were imaged and compared to the control needle without sheath. The control needle and the Sono-Sheath™ prototypes are all intended for use during a liver biopsy.

### Image Acquisition Protocol

Ultrasonography was performed using a Toshiba Aplio XG SSA-790A ultrasound machine with a 3.5 MHz curvilinear transducer (PVT-375 BT) (Canon Medical Systems Europe B.V., Zoetermeer, The Netherlands). Ultrasound settings were kept constant between measurements except for the beam focal zone. The focus was manually set to the depth of interest (5 or 10 cm). Needles were manually inserted at angles of 30° and 60° with reference to the probe at a depth of 5 cm. Due to the small size of the cadaver’s liver, 10 cm depth was studied only at a 60-degree angle. For each new needle insertion, a different area of the liver was used to prevent needle tracts, caused by previous needle insertion, influencing the needle visibility. Angles and depth were monitored using the ultrasound machine’s built-in scale. The ultrasound probe was oriented to capture the maximum portion of the needle shaft. Once the probe was optimally aligned, a 30 s clip was captured for later analysis. Multiple clips were captured on the same needle, and the optimal footage was selected for qualitative analysis by JH. Optimal footage contains the needle during insertion into the liver, the tip reaching the predefined depth, and removal of the needle. Some footage only contained a part of the insertion or removal of the needle, and some footage was taken in a wrong plain. These clips were considered not appropriate for qualitative analysis. For quantitative assessment, single images from these clips were selected by JH and JF in mutual consensus. The images selected were required to show the needle in the predefined angles and depths and in the correct plane.

### Phantom Testing

Because the sheaths could not be tested for 10 cm depth in 30° due to liver size restrictions and to obtain quantitative results under fully controlled conditions regarding insertion angle and depth, additional scans were made in a phantom consisting of a standardized echogenic fluid (CIRS, Norfolk Virginia, USA, product number 1628–02). This fluid has the same ultrasound properties as human liver tissue with respect to speed of sound, attenuation, and backscatter. The ultrasound probe and needle were fixed and images were acquired with the needle at two different depths (5 and 10 cm) and a range of angles (25°–65°) with the needle tip centered in the B mode image.

### Qualitative Evaluation

All captured ultrasound clips from the cadaveric experiment were 30 s in duration and were presented individually and randomly to five radiologists using the same reporting stations that are used for clinical care. All radiologists had a minimum 5-year experience with US-guided PLB. They were blinded for the used needle type and allowed to view clips multiple times. After the clip was reviewed, the radiologists were asked to rank each needle for needle tip visibility, needle shaft visibility, nuisance of artifacts, the brightness of the shaft compared to background, shaft delineation and if they feel confident to take a biopsy considering the visibility of the needle on a 5-point Likert scale. See Appendix A for the ranking questionnaire.

### Quantitative Evaluation

**Fig. 2:**
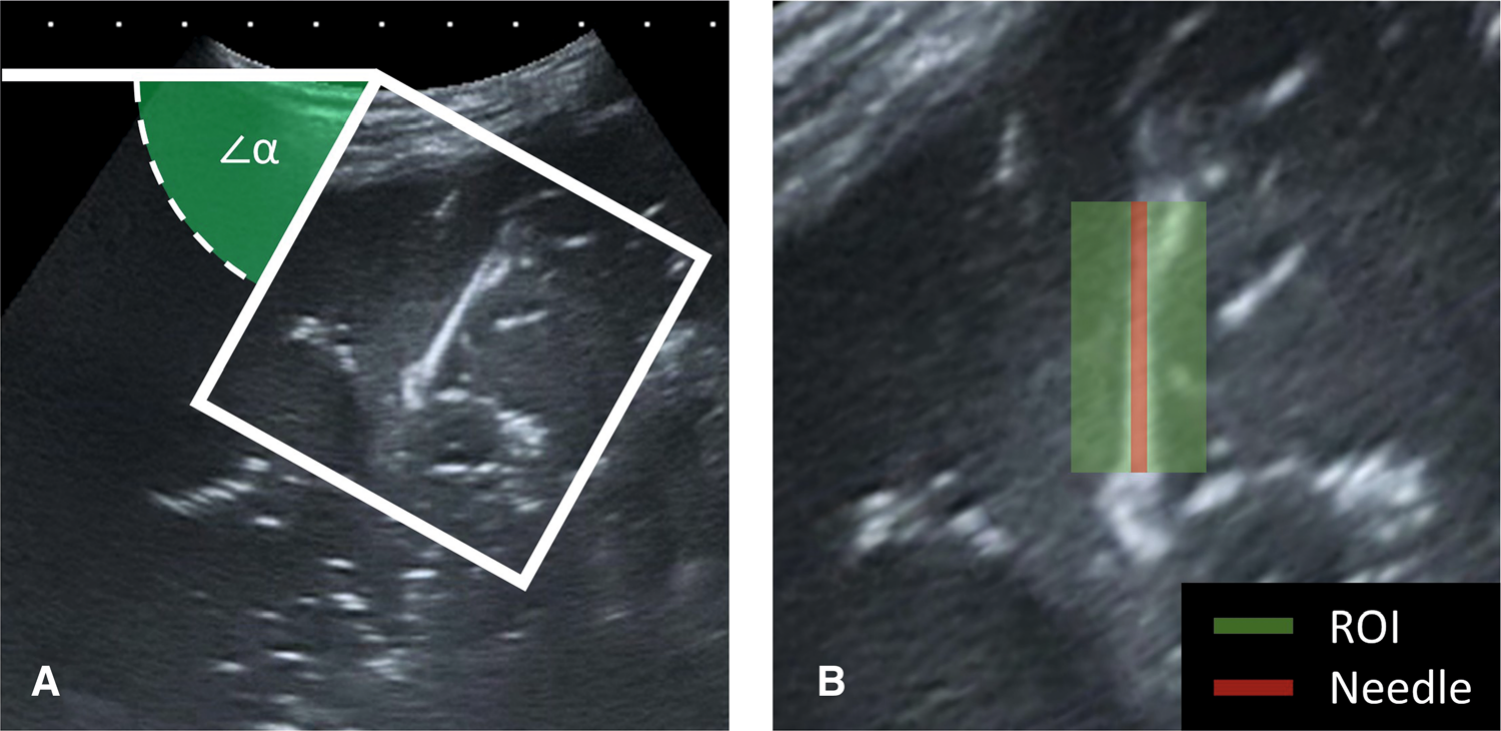
Illustration of ROI selection (caption) **A** Original US image, angle α represents the angle of rotation to obtain image B. **B** Definition of the ROI, in green the full ROI of size 2 × 1 cm, with the three center pixel columns defined as needle segment in red

To objectively quantify the ultrasound visibility of the control needle and three sheaths, a contrast analysis was performed. By analyzing a region of interest (ROI) the echogenic intensity of the needle and the contrast between the needle and the surrounding tissue could be determined. As a first step, a ROI was defined as a rectangular area of 1 cm by 2 cm. The ROI was aligned with the needle insertion angle by rotating the image after which the ROI is centered over the needle (Fig. 2.). The three center columns of pixels of the ROI were considered to contain only needle pixels and represent a segment of 1 mm in width and 20 mm in length. The insertion angle (α) was measured in the ultrasound image and is considered the true insertion angle. Also, the depth range where the ROI was placed was noted. Three metrics were calculated within the ROI: (1) the mean ± standard deviation (SD) of the echogenic intensity (EI), defined as the brightness expressed in pixels 0–255, from dark to light; (2) The mean needle-tissue contrast, defined as echogenic intensity of the needle—the intensity of surrounding tissue above the needle; and (3) Normalized EI profiles over the needle. For the phantom experiment, the same quantitative analysis was performed. All processing steps were performed using ImageJ 1.52a (U.S. National Institutes of Health, Bethesda, MD, USA) [10] software.

### Statistical Analysis

Descriptive data were presented in median and range for Likert-scale results. For the contrast analysis, values of echo intensity were described in mean and standard deviation.

Spearman's rank correlation coefficient was calculated to evaluate possible correlations between ranking and needle type. Interrater reliability was analyzed and expressed in mean agreement fraction and mean Cohen’s kappa (κ) [11]. For the calculation of *κ*, ranking scores were dichotomized with Likert-scale scores 1–3 as 0 and 4–5 as 1. A one-way ANOVA with Bonferroni correction was performed to assess for significant differences between groups concerning mean EI. *P-*values < 0.05 were considered statistically significant. Data were analyzed using SPSS version 25 (SPSS Inc., Chicago, IL, USA).

## Results

In total, twelve ultrasound videos were analyzed. The control needle and three prototypes were evaluated at a depth of 5 cm at an angle of 30° and 60°, and a depth of 10 cm at an angle of 60°. In addition, eight ultrasound videos were acquired in a phantom model and quantitatively analyzed.

### Qualitative Assessment—Ranking by Radiologists

All five radiologists completed the ranking questionnaire. The mean observer agreement fraction was 0.75 ± 0.26, which corresponds with a interrater reliability of 75%. The mean κ was 0.21 (95% Confidence Interval 0.14–0.28), which indicates a fair agreement among radiologists.

**Fig. 3:**
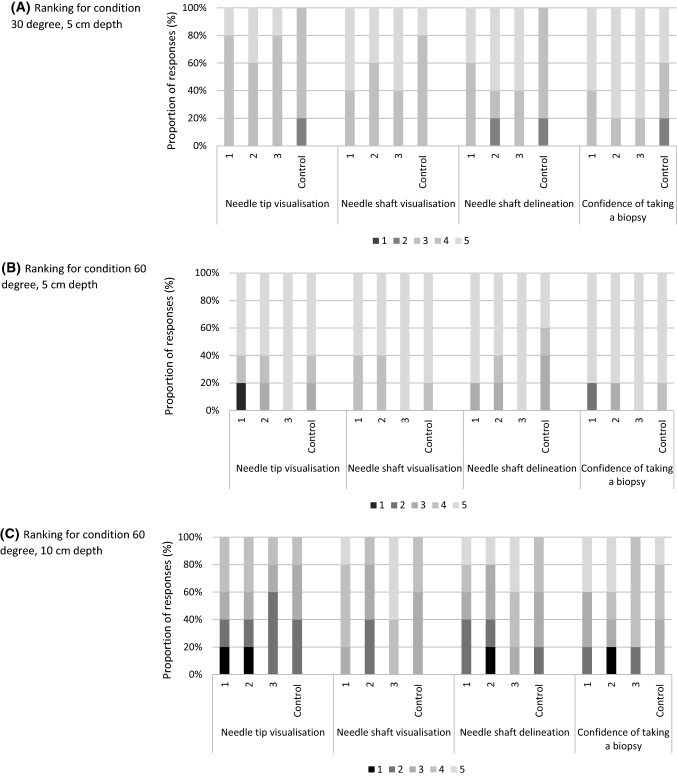
Observer Frequency plots per condition

In general, the needle and all sheaths were ranked lower at a 10 cm depth compared to 5 cm depth. At a depth of 10 cm the median rank for the prototypes 1, 2, 3 and the control needle were 3, 3.5, 4 and 3, respectively, while in the condition for 5 cm, all sheaths were ranked with a median of 5 and for the control with 4.5. In addition, all sheaths and the needle were rated lower at a 30° angle than at 60°. The three prototype echogenic sheaths were ranked higher than the control needle at 30° with 5 cm depth, with equal performance for the other conditions (Fig. 3). At 30°, the radiologists expressed more confidence in taking a biopsy with the sheaths than with the control needle, with a median of 1 Likert score difference. In all conditions, sheath 3 was ranked higher than the control needle. At 5 cm depth at 30°, sheath 3 was unanimously ranked highest with a median score of 5. It also received the highest rank at 10 cm at 60°, with a median score of 4. A Spearman’s correlation test between total needle rank (median) and needle type showed a positive correlation coefficient of 0.402, which was not statistically significant (p = 0.195).

### Quantitative Assessment—Contrast Analysis

#### Echogenic Intensities in the Cadaver Liver

The measured average needle insertion angles were 37° ± 4, 62° ± 7 at the intended 5 cm depth, and 67° ± 10 at 10 cm depth. The depths at the center of the ROIs ranged from 2.9–4.0 cm and 5.1–8.0 cm for the 5 cm and 10 cm depth approximations respectively. Table 1 shows the mean echogenic intensity (EI) levels of control and prototypes, and the contrast between the needle or prototype and the surrounding tissue. At 10 cm depth, sheath 1 and 2 show a lower peak intensity than sheath configuration 3 and the control needle. However, sheath 1 and 2 were also scanned considerably deeper and with a greater insertion angle compared to sheath 3 and the control needle, as can be seen in Table 1.

**Table 1:** Contrast analysis per needle and condition (caption) For each needle configuration and setting, the depth of the center of the ROI, insertion angle, needle echo intensity and contrast are given. The contrast is defined as the difference between the mean EI of the needle and the mean EI of the tissue above the needle

Needle config #	Depth of center of ROI (cm)	Angle (°)	Mean needle EI (SD)	Contrast
Sheath 1	3.4 3.2	43 70	144 (23) 177 (19)	69 104
	8.0	66	94 (23)	37
Sheath 2	3.3 3.7	36 62	149 (29) 197 (23)	98 110
	7.5	83	103 (31)	49
Sheath 3	3.3 3.5	33 63	193 (22) 211 (14)	135 116
	6.3	65	192 (11)	115
Control	2.9 4.0	37 51	176 (33) 141 (37)	102 57
	5.1	55	163 (25)	91

#### Echogenic Intensities in the Phantom Liver

**Fig. 4:**
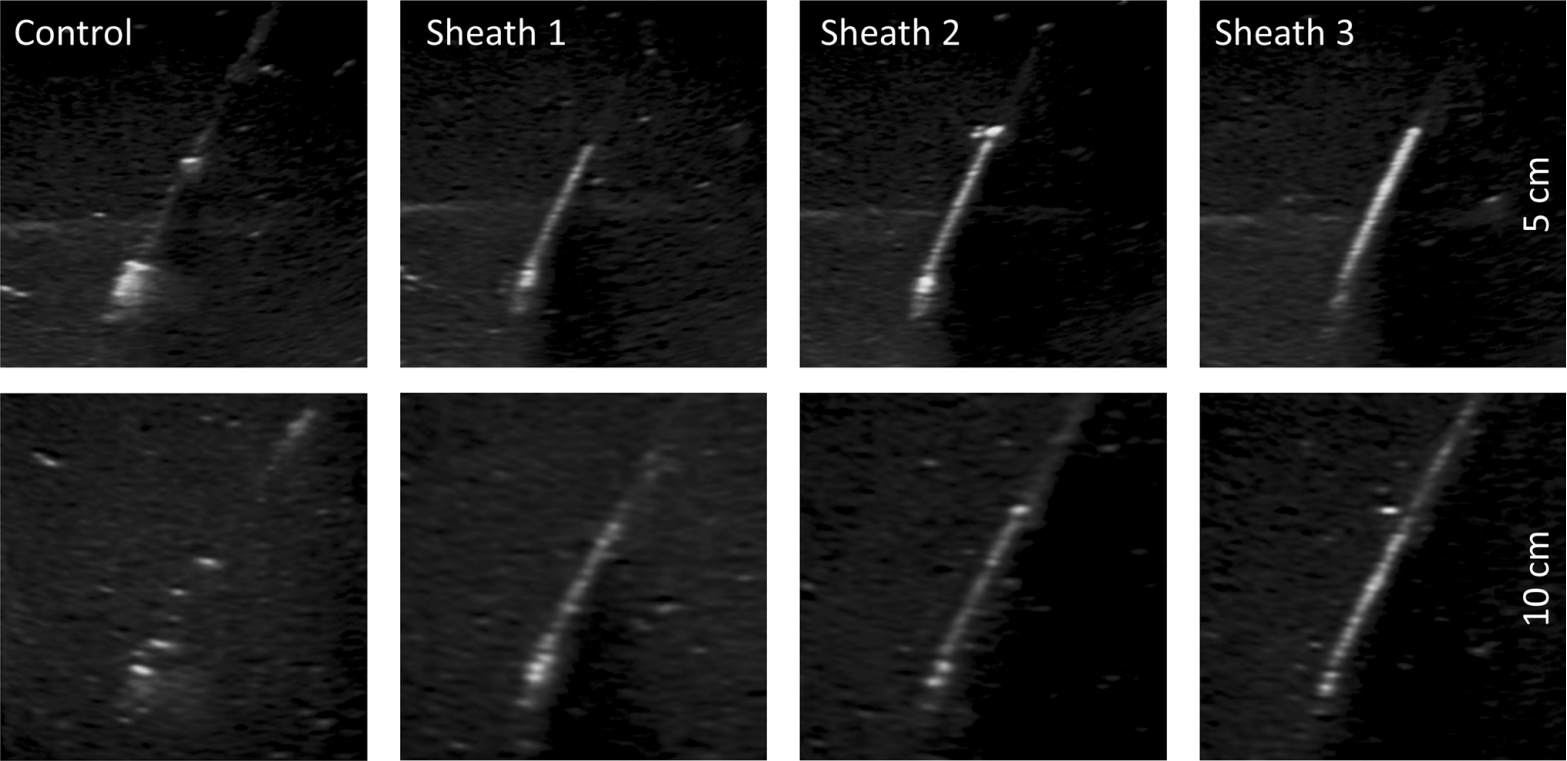
Control and Study sheaths displayed in a phantom model in fixed conditions (65° at 5 and 10 cm)

**Fig. 5:**
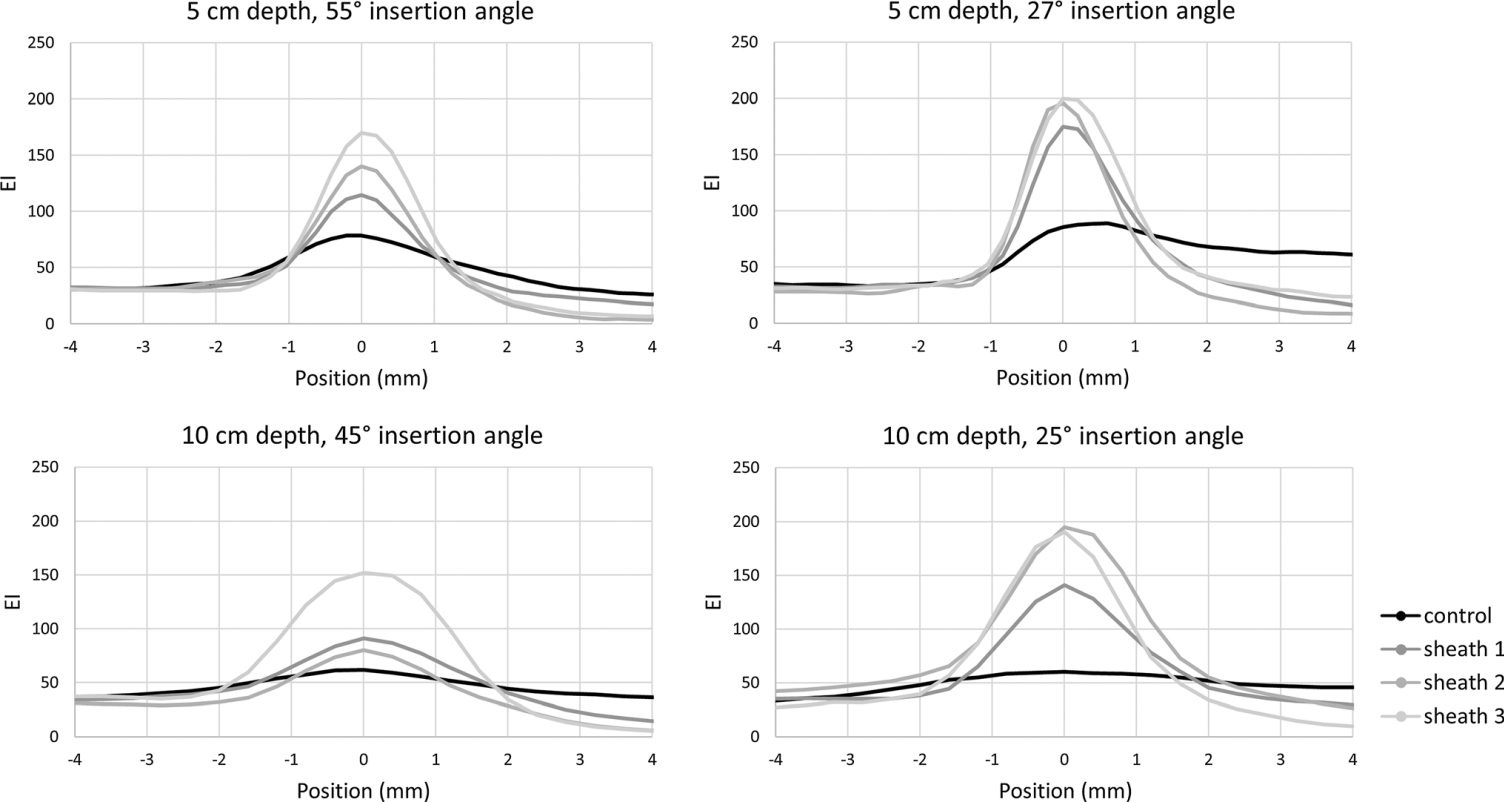
Echogenic intensity (EI) profiles for control and sheaths in all tested conditions

The images acquired from the phantom model demonstrate different appearances of the needle tips at fixed insertion angle and depth. Figure 4 shows an increasingly brighter appearance with increasing sheath number, especially at larger depths. Figure 5 shows the corresponding EI profiles. In all conditions, the sheaths show a higher and narrowed peak of EI profiles compared to the control needle. This means that the signal is brighter and has a stronger contrast near the edges of the needle when using a sheath.

The one-way ANOVA showed a statistically significant effect of a sheath *F* (3,12) = 7.78, *p* = 0.004. Post hoc analysis showed that mean echogenic intensities for sheath 2 and 3 were significantly higher than the control, with a mean difference of 97 for sheath 2 (*p* = 0.016, 95% CI 16–177) and a mean difference of 115 for sheath 3 (*p* = 0.005, 95% CI 34–195). For sheath 1, a marginally statistically significant difference with the control was observed (mean difference 77, *p* = 0.064, −3–157).

**Fig. 6:**
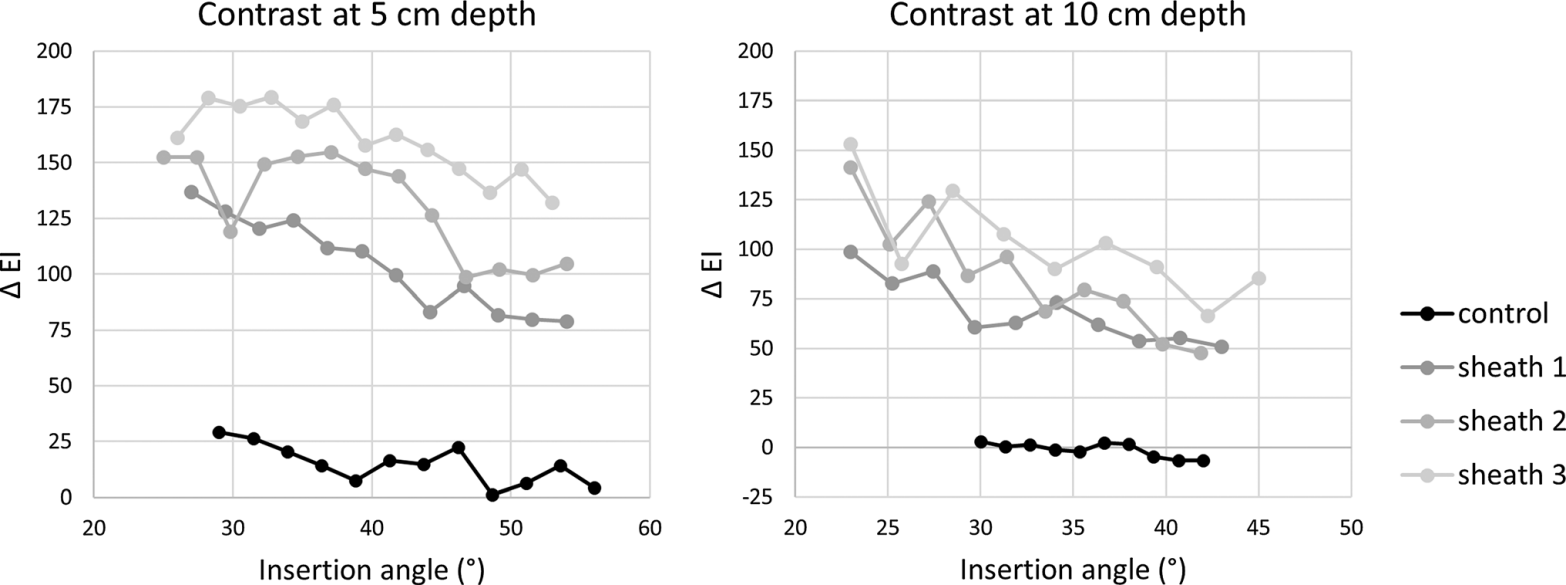
Contrast values for control and sheaths in all tested conditions

Concerning contrast, all sheaths showed higher contrast values with respect to the control needle (Fig. 6). At 10 cm depth, the contrast of the control needle even reaches an EI value of zero, whereas all sheaths have contrast values of 50 to 150 EI units for all measured angles.

## Discussion

In this pilot study, we have evaluated a new echogenic sheath in a human cadaver liver and phantom model. Qualitative assessment showed that sheath 3 consistently outranked the control needle. In addition, at an insertion angle of 30° the radiologists expressed more confidence in taking a biopsy with the echogenic sheaths than with the control needle. Quantitative assessment showed favorable intensity profiles for the prototypes, especially for sheath 3. In the phantom experiment, the effect of a sheath was significant. Sheaths 2 and 3 showed significantly better echogenic intensity profiles than the control needle for all angles and depths.

Needle visibility was ranked higher at 60° than at 30°, contradicting literature that describes decreased needle visibility as needle angles increases [4, 5, 12, 13]. The same ranking is observed for the sheaths, even though they are designed to maintain good visibility, regardless of the insertion angle. A possible explanation for our result could be found in the alignment of the needle compared to the transducer in the 30° condition. Because the angle was less steep and we desired to visualize the shaft as well, the tip might have been depicted more in the periphery of the image and less central relative to the central beam of the transducer. Perhaps this had led to a relative lower ranking by the radiologist, as they are used to a more centralized depiction of the needle tip.

Visualization of the needles became suboptimal in deeper insertions or when the background was more echogenic, which is in line with other studies [14]. Though the cadaver liver in our study did not have an abundance of fatty tissue (steatosis), we did encounter areas with air and highly echogenic wall structures surrounding the liver. This aspect accounts for the largest limitation of this study. Even though a cadaver is considered an appropriate preclinical model for new surgical innovations, this may be different for innovations studied by ultrasonography. As seen in this study, we encountered several areas with air present around the liver. This was also confirmed by some of the radiologists who mentioned that parts of the cadaver liver had a considerably different appearance from an in vivo human liver. Even after careful selection of areas with homogeneous liver tissue, the presence of air in the liver could have had an influence on needle echogenicity, by for instance air bubbles getting caught in the needle trajectory.

A second limitation is that despite our condition settings of 30° and 60° and predefined depths in the cadaver liver, we retrieved data in conditions that can be considered an approximation of these predefined circumstances. Especially, the deeper measurements of 10 cm were more difficult to obtain in the cadaveric liver and as a result varied between needle and prototypes. In the phantom, however, this did not pose a problem and the predefined conditions were easy to achieve. A final limitation concerns that qualitative and quantitative analyses were all performed using one single clip or still image of each prototype.

Despite these limitations, we consider the cadaver model as the model that best resembles the in vivo situation when compared to optimally controlled simulations based on phantoms. The lack of control and limitations in US imaging in cadaver studies are similar to the in vivo situation by means of anatomical barriers limiting the options to optimally visualize the needle. A second strength is that both a ranking assessment was performed (subjective measure) as well as a contrast analysis (objective measure), allowing for a full, comprehensive assessment of the sheaths.

This pilot study suggests placing an echogenic sheath over a regular biopsy needle during ultrasonography is feasible and could be of considerable advantage in increasing needle visibility. Sheath 3 consistently outranked the others and had significantly higher echogenic intensity profiles than the control needle, without appearing to give artifacts or shadowing. However, to fully understand the benefit of a polymeric sheath in PLB and to assess its safety, further studies in in vivo models and a clinical study are required.

## Appendix

**Sono-Sheath™ Questionnaire.**

**Observer: _________________.**

**Needle code: ______________.**

**Table:**

Criteria	Ranking				
Needle tip visualization	1 No needle visible	2	3	4	5 Excellent Visibility
Needle shaft visualization	1 No shaft visible	2	3	4	5 Excellent Visibility
Nuisance of artifacts	1 Significant number of artifacts	2	3	4	5 No artifacts
Brightness of the shaft	1 Not distinguishable from the background	2	3	4	5 Very Bright
Delineation/sharpness of the shaft	1 Ill defined	2	3	4	5 Very defined
Considering the needle visibility, do you feel confident enough to take a biopsy?	1 Absolutely not	2	3	4	5 Absolutely

1 = Strongly disagree, 2 = Disagree, 3 = Neutral, 4 = Agree, 5 = Strongly agree.
